# The relationship between air pollutants and preterm birth and blood routine changes in typical river valley city

**DOI:** 10.1186/s12889-024-19140-2

**Published:** 2024-06-24

**Authors:** Jimin Li, Jiajia Gu, Lang Liu, Meiying Cao, Zeqi Wang, Xi Tian, Jinwei He

**Affiliations:** Medical School of Yan’an University, Yan’an, Shaanxi, China

**Keywords:** Air pollution, Preterm birth, Blood cell

## Abstract

**Objective:**

To collect maternal maternity information on preterm births in two tertiary hospitals in the urban area of Baota District, Yan'an City, from January 2018 to December 2020, to explore the long-term and short-term effects of air pollutants (PM_2.5_, PM_10_, SO_2_, NO_2_, CO and O_3_) and preterm births, and to explore changes in blood cell counts due to air pollutants.

**Methods:**

Daily average mass concentration data of six air pollutants in the urban area of Yan'an City from January 1, 2017 to December 31, 2020 were collected from the monitoring station in Baota District, Yan'an City. Meteorological information was obtained from the Meteorological Bureau of Yan'an City, including temperature,relative humidity and wind speed for the time period. The mass concentration of air pollutants in each exposure window of pregnant women was assessed by the nearest monitoring station method, and conditional logistic regression was used to analyze the relationship between air pollutants and preterm births, as well as the lagged and cumulative effects of air pollutants. Multiple linear regression was used to explore the relationship between air pollutants and blood tests after stepwise linear regression was used to determine confounders for each blood test.

**Results:**

The long-term effects of pollutants showed that PM_2.5_, PM_10_, SO_2_, NO_2_and CO were risk factors for preterm birth. In the two-pollutant model, PM_2.5_, PM_10_, SO_2_ and NO_2_ mixed with other pollutants were associated with preterm birth. The lagged effect showed that PM_2.5_, PM_10_, SO_2_, NO, and CO were associated with preterm birth; the cumulative effect showed that other air pollutants except O_3_ were associated with preterm birth. The correlation study between air pollutants and blood indicators showed that air pollutants were correlated with leukocytes, monocytes, basophils, erythrocytes, hs-CRPand not with CRP.

**Conclusion:**

Exposure to air pollutants is a risk factor for preterm birth. Exposure to air pollutants was associated with changes in leukocytes, monocytes, basophils and erythrocytes and hs-CRP.

**Supplementary Information:**

The online version contains supplementary material available at 10.1186/s12889-024-19140-2.

## Introduction

While cities in China have been accelerating the progress of urbanization in recent years, a large amount of industrial emissions and vehicle exhausts have polluted the air. Air pollution mainly includes particulate matter (PM_2.5_, PM_10_), ozone (O_3_), nitrogen dioxide (NO_2_) and sulphur dioxide (SO_2_). A large number of studies in various countries have shown that air pollutants are associated with a variety of systemic diseases in the human body [1, 2].such as the nervous system [3],the immune system [4],the endocrine system [5]and the reproductive system [6]. Air particulate matter can enter the body and cause direct damage to the respiratory tract [7]. Long-term exposure to ozone pollution may lead to airway inflammation and decreased lung function [8],and eye irritation [9]. In addition, ozone has been associated with an increased incidence of cardiovascular disease [10], which has also been associated with increased mortality from diseases of the cardiovascular system [11]. It has also been shown that prolonged exposure to high mass concentrations of nitrogen dioxide and sulfur dioxide can also cause respiratory irritation [12]. Exposure to nitrogen dioxide and sulfur dioxide is also associated with cardiovascular disease [13]. In addition to the long-term exposure effects of pollutants, short-term exposure also has an impact on the human body. Studies have shown that short-term exposure to air pollutants is not only associated with ischemic stroke [14], but also associated with childhood respiratory diseases [15]. Short-term exposure has also been documented to cause changes in male reproduction-related hormones [16]. Even anxiety, depression, mental illnesses [17], mortality rate [18] have also been shown to be associated with short-term exposure.

Air pollutants have also been shown to be associated with adverse pregnancy outcomes [19]. Preterm birth is one of the adverse pregnancy outcomes and it is also one of the most common perinatal complications in pregnant women, and according to statistics, 15 million preterm babies are born worldwide every year [20]. With the increasing social pressure, environmental and climate changes and the full opening of the two-child policy, China's preterm birth rate is increasing year by year [21]. Preterm babies are often born with preterm complications and are at increased risk for other diseases as they grow. Preterm birth and its complications are the leading cause of neonatal deaths. Pregnancy is a long and multifactorial process, so there are many factors that can lead to preterm birth in pregnant women. Common factors that lead to preterm birth are the pregnant woman's own factors, genetic factors, and infections during pregnancy, environmental factors, psycho-behavioral factors  and ethnic factors.Although the majority of studies on air pollutants and preterm birth have shown a correlation, there are inconsistencies in the major exposure windows, such as a strong correlation between exposure to PM_2.5_ and preterm birth throughout the entire pregnancy [22], and some studies suggesting that PM_2.5_ has its strongest effect in late pregnancy [23] or a correlation with exposure in the week prior to delivery [24].

Some studies have also shown a correlation between blood routine and preterm birth [25, 26]. Blood routine examination is a test to judge blood conditions and diseases by observing the changes in the number and morphological distribution of blood cells. Changes in blood cell counts reflect subtle changes in the body.

Existing studies have also shown that air pollutants have a certain effect on blood. It has been shown that exposure to air pollutants decreases the number of red blood cells and increases the ratio of white blood cells, neutrophils and lymphocytes, with no effect on monocytes [27]. It has also been shown that increased PM_2.5_ mass concentrations are associated with lower erythrocyte [28]. C-reactive protein(CRP) is a nonspecific marker of inflammation and tissue damage in the human body, hypersensitive C-reactive protein(hs-CRP) is synthesized by the liver and is a nonspecific marker of the acute phase of the systemic inflammatory response. Changes in the concentrations of both have been shown to be associated with a variety of human systemic diseases [29, 30]. However, there are still some discrepancies in the studies on blood markers [31, 32], Therefore, further research is needed on air pollutants and blood markers.

The city of Yan'an is located in the hilly and gully area of the Loess Plateau in northern Shaanxi Province, which is a typical hilly and gully landscape. The urban area of Yan'an is located in the middle of a "Y" shaped valley, and the narrow geographic environment facing the mountains on both sides creates a mountain screen effect, which restricts the horizontal diffusion of pollutants in the near-surface layer, and then creates a buildup of pollutants in the air above the city. Although air quality in Yan'an has improved year by year in recent years, there are still periods of time when air pollutant mass concentrations are high.

In this study, we collected data on pregnant women and air pollutants in the urban area of Yan'an City from 2018 to 2020 to assess the exposure dose of air pollutants received by pregnant women during pregnancy. The relationship between air pollutants and preterm birth and the relationship between air pollutants and blood indicators were analyzed to provide a basis for the impact of air pollution on preterm birth. Based on the existing literature we predict that atmospheric pollutants may be a risk factor for the occurrence of preterm birth in pregnant women. At the same time, air pollutants may also cause certain changes in the blood counts of pregnant women.

## Methods

### Research population

In this study, the data of pregnant women in the Department of Obstetrics and Gynecology of two local hospitals in Bota District, Yan'an City, were collected from 2018 to 2020. After collecting data information, we confirmed the inclusion and exclusion criteria. Inclusion criteria: residents of Baota District, Yan'an City, who have lived in the district for one year or more; normal mental status; no communication barriers; no assisted conception; no missing information; no major diseases.Exclusion criteria: people with various missing information; people with abnormal mental status who cannot communicate; people with assisted conception or multiple pregnancies; people with hereditary diseases.All pregnant women have signed an informed consent form, and the study has been approved by the ethics committee of Medical School of Yan'an University (approval number: 2018051).

After screening for inclusion criteria, 460 cases of preterm birth(PTB) with complete maternal data were collected as the case group. In order to reduce the influence of confounding factors on the results of the study, we took term births(TB) of the same age and the same gestation as preterm births as the control group and selected 1,840 cases of term births as the control group in a ratio of 1 to 4. The data collected from pregnant women included the following: general data of pregnant women (name, age, gestational address, occupation), pregnancy data (gestational age, Pregnancy times, number of births, number of caesarean sections, regularity of menstrual cycle, season of the last menstrual period, Complications diseases, Comorbidity diseases, and hypertension in pregnancy), neonatal data (birth weight, date of birth) and routine blood data:leukocyte count (WBC), percentage of neutrophils (NEUT), percentage of lymphocytes (LYM), percentage of monocytes (MONO), percentage of eosinophils (EOS), percentage of basophils (BAS), erythrocyte count (RBC), hs-CRP,CRP.

Complications diseases: severe vomiting of pregnancy, ectopic pregnancy, placenta previa, placental abruption, excessive or low amniotic fluid, premature rupture of membranes, hyperemesis gravidarum, acute chorioamnionitis.

Comorbidity diseases: combined cardiovascular diseases (congenital heart disease, rheumatic heart disease,etc.), combined hematological diseases (chronic aplastic anemia, idiopathic thrombocytopenic purpura,etc.), combined respiratory diseases (tuberculosis, bronchial asthma), combined gastrointestinal system diseases (viral hepatitis, acute appendicitis,etc.), combined urinary system diseases (acute pyelonephritis, chronic glomerulonephritis,etc.), combined endocrine system diseases (hyperthyroidism, hypothyroidism,etc.), combined dermatological disorders (scleroderma, hives, herpes), combined infectious diseases (cytomegalovirus infections, genital herpes,etc.), combined tumors (uterine fibroids, cervical cancer,etc.).

### Pollutant exposure assessment

Data sources for air pollutants: All pollutants information was obtained from the Qingyue Open Environmental Data Centre (http://data.epmap.org/), and data were obtained from the daily average mass values of six pollutants (PM_2.5_, PM_10_, SO_2_, NO_2_, CO and O_3_) at four monitoring stations in Baota District, Yan'an City (the mass concentration unit of CO is mg/m^3^, and the mass concentration of the remaining air pollutants is μg/m^3^). Meteorological data were obtained from Yan'an Meteorological Monitoring Station. The time span of pollutant data and meteorological data is from 1 January 2017 to 31 December 2020.

**Fig. 1:**
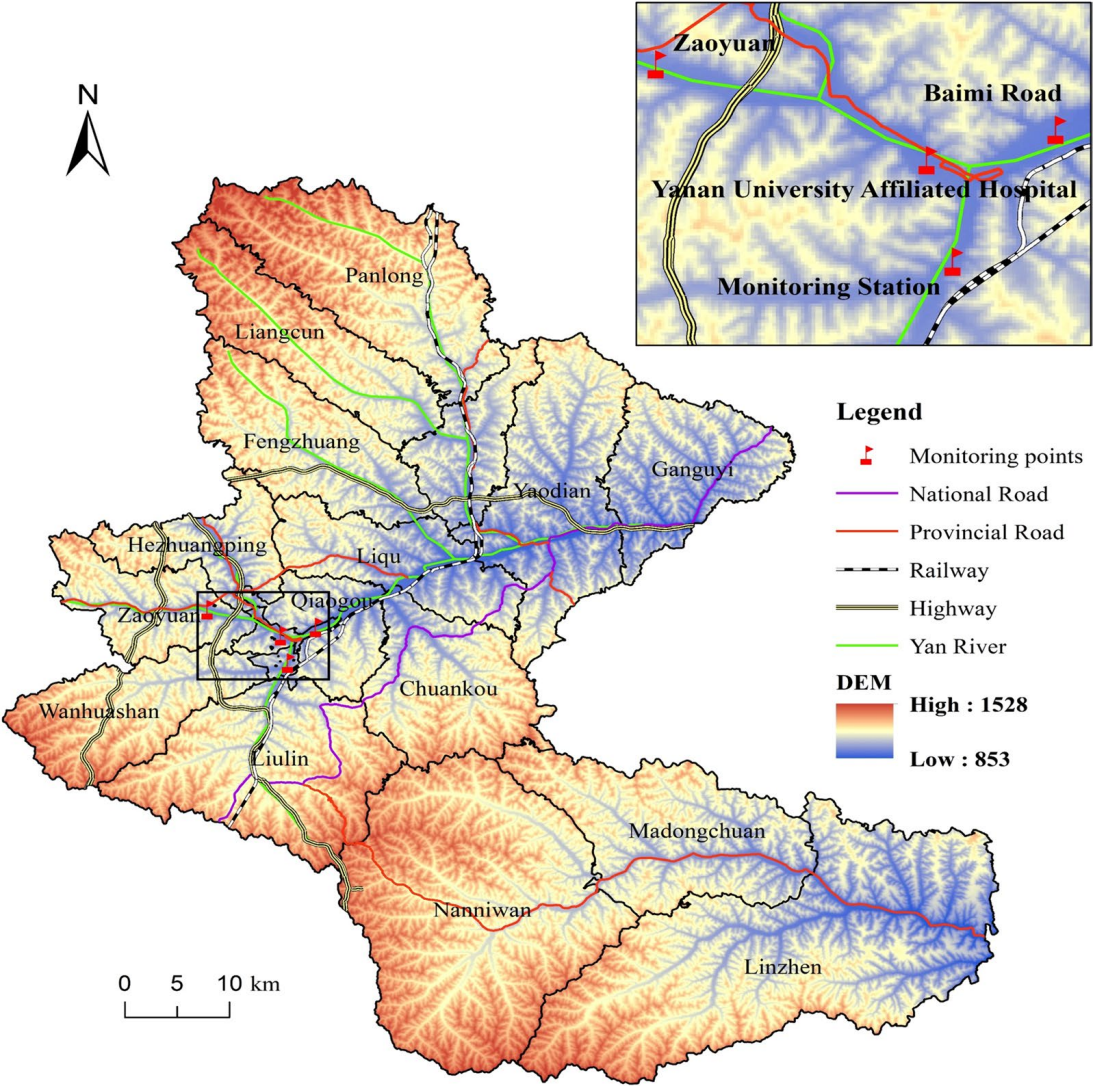
Location of Yan'an city in the surrounding area

Exposure assessment method: Based on the address of the pregnant woman's place of residence during pregnancy, the latitude and longitude of her place of residence were obtained from Gaode Map. The distance from the latitude and longitude of the place of residence to the latitude and longitude of the monitoring station was calculated, and the pollutant mass concentration of the nearest monitoring station to the place of residence was selected as the individual's exposure mass concentration. After dividing each exposure window according to the date of the last menstrual period of pregnant women, the mass concentration of pollutants in each exposure window was calculated (Fig. 1).

#### Calculation of pollutants

 The exposure window is divided according to the date of the mother's last menstrual period into:E: the entire pregnancy (last menstrual period to the date of birth of the births), T_1_:early pregnancy (last menstrual period to the twelfth week of gestation), T_2_:mid-pregnancy (the thirteenth week of gestation to the twenty-seventh week of gestation), and T_3_:late pregnancy (the twenty-eighth week of gestation to the date of birth of the births). Considering that air pollutants have short-term effects in addition to long-term effects on birth, lagged and cumulative effects were calculated based on the date of admission to hospital. Matching of same-day and lag-day mass concentrations: Short-term same-day lagged mass concentrations of each pollutant on the day of the admission date and before the admission date are matched based on the maternal admission date. This exposure mass concentration is the daily average of the day, where the short-term lagged exposure dates are the day of the admission date (Lag0) and the days 1 (Lag1), 2 (Lag2), 3 (Lag3), 4 (Lag4), 5 (Lag5), 6 (Lag6), and 7 (Lag7) before the admission date. Calculation of mass concentration values for cumulative exposure: Matching the mass concentration of each pollutant prior to the date of admission to the date of maternal admission, the cumulative exposure mass concentration value is the average of all daily averages over the exposure period. The cumulative exposure period is 1 day (Lag-1), 2 days (Lag-2), 3 days (Lag-3), 4 days (Lag-4), 5 days (Lag-5), 6 days (Lag-6), and 7 days (Lag-7) before the day of admission.

### Statistical methods

A database was created by EXCEL and data were analyzed by IBM SPSS 20. Normality test for pollutants was performed and pollutants were described using mean, median, standard deviation(SD), and interquartile range(IQR). The categorical variable information in the general information of pregnant women was statistically described using frequency (n) and composition ratio (%), and the difference between the case and control groups was compared using the chi-square test to determine confounders in turn. The correlation between air pollutants and temperature and relative humidity was analyzed, and pollutant mass concentrations were calculated for each exposure window using the R language. The long-term effects and lagged and cumulative effects of air pollutants and preterm birth were analyzed using conditional logisitic models, and after adjusting for each confounding factor, pollutants were introduced into the conditional logistic model with as a continuous variable with in, and ORs and 95% confidence intervals were calculated. After identifying the association between air pollutants and preterm birth, we adjusted for confounders and investigated the relationship between air pollutants and blood counts using multiple linear regression. Logistic regression was then used to explore the relationship between air pollutants and C-reactive protein at a test level of 0.05 (two-sided test).

#### Correlation analysis

Spearman's correlation analysis was used to analyze the correlation between air pollutants, blood markers, and meteorological factors (mean temperature throughout the pregnancy, relative humidity throughout the pregnancy, and mean wind speed throughout the pregnancy).

#### Sensitivity analysis

In order to verify the stability of the main model, the effect of each pollutant on preterm birth was analyzed separately. Then other confounding factors were added one by one to analyze the relationship between each pollutant and preterm delivery. Three sensitivity analyses were performed with meteorological factors as confounding factors: (1) Model 1: correlation analysis between a single pollutant and each blood index; (2) Model 2: add occupation, birth order and last menstrual season to analyze the correlation between each pollutant and preterm delivery in Model 1; and (3) Model 3: add menstrual cycle and complications diseases to Model 2.

## Results

### General situation of pollutants in urban areas of Baota District

**Fig. 2:**
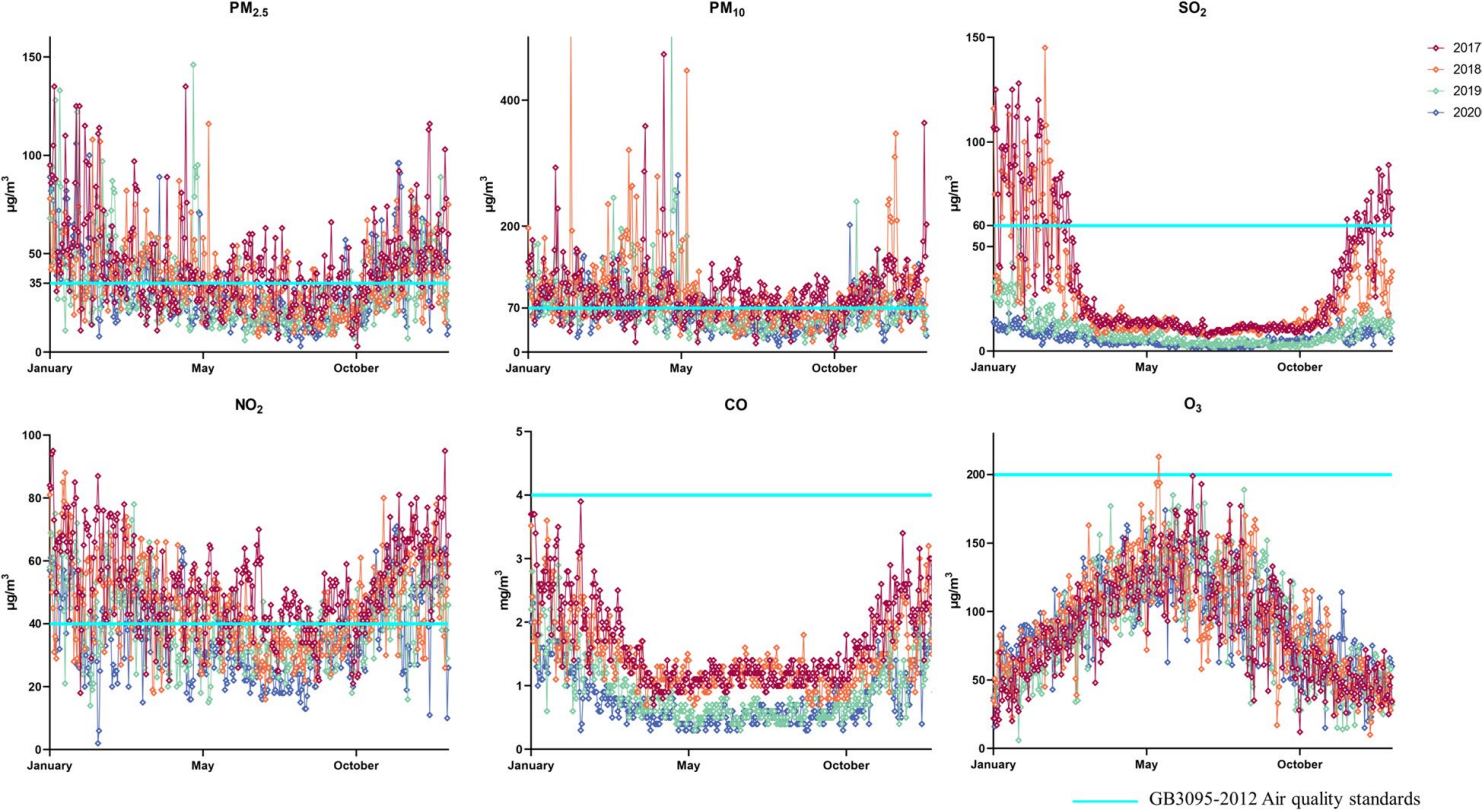
General Air Quality in the Baota District, 2017–2020

Mass concentration data of six pollutants in Yan'an City were collected from 2017 to 2020.PM_2.5_, SO_2_, NO_2_ and CO showed a U-shaped trend, while O_3_ an inverted U-shaped pattern.The mass concentration of SO_2_ was basically flat from March to October each year, and then increased sharply from November to February of the following year.From April to October, there was little change in the mass concentration of CO, and then increased and decreased from November to March of the next year. CO mass concentrations also varied slightly from April to October, then increased and decreased from November to March, and O_3_ mass concentrations increased gradually from January to April, reached a maximum in May–June, and then decreased over time. Mass concentrations of all six pollutants showed seasonal variations, with O_3_ increasing in the spring and summer compared to fall and winter,peaking between May and June (Fig. 2).

**Fig. 3:**
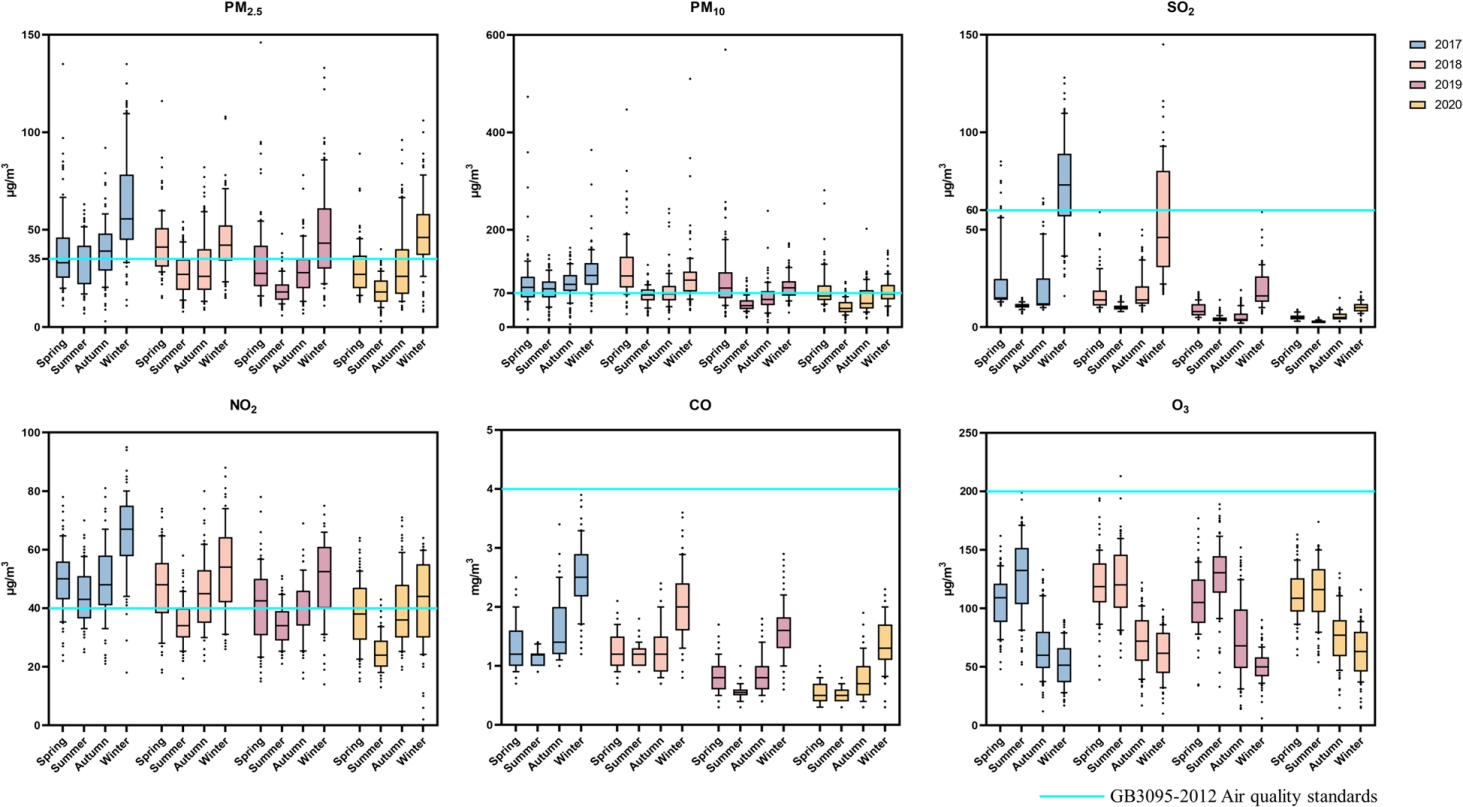
Air Quality Statistical Indicators for Baota District, 2017–2020

PM_2.5_, PM_10_, SO_2_ and NO_2_ were all above the national secondary standard at some time during each year. The median and maximum mass concentration of PM_2.5_ in winter are higher than the secondary standard, indicating that the pollution is mainly concentrated in winter. The PM_2.5_ mass concentration is gradually decreasing with the change of time. Compared with other air pollutants, Mass concentrations of PM_10_ are concentrated near the secondary standard. The mass concentration of PM_10_ also shows a decreasing trend from year to year. SO_2_ has been below the state's secondary standard since 2019. Mass concentrations of NO_2_ were above the secondary standard at times in each year and again showed higher concentrations in winter and spring than in summer and fall. CO and O_3_ are generally within the state's secondary standard mass concentrations (Fig. 3).

### General demographic characteristics of the study population

Based on Table 1, the chi-square analysis of the study population showed statistically relevant differences between the case and control groups in terms of maternal occupation, number of births, season of last menstruation, menstrual cycle, fetal birth weight, complications diseases, and hypertension in pregnancy. In the collected study population, pregnant women who experienced preterm birth relative to term births had a higher proportion of those who were 25–30 years of age, unemployed, > 1 times pregnancy, ≤ 1 times births, ≤ 1 times caesarean sections, last menstrual season of winter, regular menstrual cycle, fetal birth weight < 2500, and suffered from complications diseases, Comorbidity diseases, no hypertension in pregnancy. The greater proportion of preterm births occurring in winter is also consistent with the seasonal variation of air pollutants (Table 1).

**Table 1:** Chi-Square test for data on pregnant women

	TB		PTB		𝜒^2^	P
	n	%	n	%		
Age						
< 20	28	1.52	7	1.52	-	-
20-	236	12.83	59	12.83		
25-	700	38.04	176	38.26		
30-	632	34.35	157	34.13		
35-	212	11.52	53	11.52		
40-	32	1.74	8	1.74		
Occupation						
National Civil Servants	194	10.54	35	7.61	12.770	0.026
Professional and technical staff	382	20.76	83	18.04		
Business and Services	14	0.77	6	1.31		
Agriculture	318	17.28	73	15.87		
Other special occupations	340	18.48	80	17.39		
Unemployed	592	32.17	183	39.78		
Pregnancy times						
1	693	37.70	173	37.60	-	-
> 1	1147	62.30	287	62.40		
Number of births						
≤ 1	1347	73.20	377	82.00	15.009	< 0.001
> 1	493	26.80	83	18.00		
Number of caesarean sections						
≤ 1	1542	83.80	398	86.50	2.058	0.151
> 1	298	16.20	62	13.50		
Last menstrual season						
Spring	1012	55.00	116	25.22	191.648	< 0.001
Summer	422	22.94	106	23.04		
Autumn	238	12.93	115	25.00		
Winter	168	9.13	123	26.74		
Menstrual cycle						
Regularity	1760	95.70	424	92.20	9.296	0.002
Irregular	80	4.30	36	7.80		
Fetal birth weight						
< 2500	48	2.60	251	54.60	878.345	< 0.001
≥ 2500	1792	97.40	209	45.40		
Complications diseases						
yes	1064	57.80	342	74.30	42.276	< 0.001
no	776	42.20	118	25.70		
Hypertension in pregnancy						
yes	248	13.50	143	31.10	80.868	< 0.001
no	1592	86.50	317	68.90		
Comorbidity diseases						
yes	942	51.20	236	51.30	0.002	0.967
no	898	48.80	224	48.70		

### Comparison of pollutants by exposure window for pregnant women

According to Table 2, throughout the pregnancy, the mean PM_2.5_, PM_10_, SO_2_, NO_2_ and CO exposure mass concentrations in the preterm pregnant women were 33.732 μg/m^3^, 77.506 μg/m^3^, 12.136 μg/m^3^, 38.084 μg/m^3^ and 0.993 mg/m^3^, all of which were higher than those in the term group. And the O_3_ mass concentration was lower than that in the term group. In early pregnancy, the mass concentrations of PM_2.5_, PM_10_, SO_2_, NO_2_ and CO were also higher in the preterm group than in the term group, with mass concentrations of 34.791 μg/m^3^, 79.017 μg/m^3^, 11.990 μg/m^3^, 38.701 μg/m^3^, and 1.023 mg/m^3^, and O_3_ mass concentrations were also lower than in the term group. In mid-pregnancy, the mass concentrations of PM_2.5_, PM_10_, SO_2_, NO_2_ and CO were 33.150 μg/m^3^, 77.283 μg/m^3^, 12.180 μg/m^3^, 37.830 μg/m^3^, and 0.978 mg/m^3^, which were also higher than those in the term birth group. And the mass concentration of O_3_ was lower than that in the term birth group. In late pregnancy, the mean values of SO_2_ and O_3_ exposure mass concentrations were higher than those of the full-term group, with mass concentrations of 12.165 μg/m^3^ and 90.232 μg/m^3^, while PM_2.5_, PM_10_, NO_2_ and CO were lower than those of the full-term group (Table 2).

**Table 2:** Comparison of statistics of the mass concentrations of pollutants by exposure window

	TB				PTB			
	Mean	Median	SD	IQR	Mean	Median	SD	IQR
E								
PM_2.5_	31.963	31.746	3.429	5.324	33.732	34.265	6.240	10.166
PM_10_	70.686	66.529	10.714	17.433	77.506	77.930	16.708	29.280
SO_2_	9.070	6.329	5.005	9.033	12.136	6.786	9.593	11.865
NO_2_	37.378	36.856	3.452	4.357	38.084	37.520	6.121	9.138
CO	0.917	0.834	0.237	0.433	0.993	0.885	0.355	0.575
O_3_	92.499	93.147	7.281	11.292	90.270	91.946	14.582	24.318
T_1_								
PM_2.5_	29.264	28.671	8.057	9.466	34.791	34.129	9.715	16.480
PM_10_	71.904	71.653	22.338	26.037	79.017	76.068	25.237	27.230
SO_2_	7.667	6.257	4.997	5.921	11.990	9.066	10.874	7.420
NO_2_	35.311	34.378	4.835	5.755	38.701	38.112	7.619	10.639
CO	0.804	0.680	0.289	0.518	1.023	1.073	0.421	0.611
O_3_	110.251	120.979	24.201	33.782	90.693	92.524	29.046	51.024
T_2_								
PM_2.5_	29.508	26.367	9.521	15.856	33.150	32.388	11.079	20.123
PM_10_	64.252	60.953	20.284	25.537	77.283	74.414	28.889	37.747
SO_2_	8.277	6.657	5.905	6.929	12.180	7.482	12.909	8.394
NO_2_	36.726	36.442	5.940	8.289	37.830	37.051	8.930	11.240
CO	0.884	0.878	0.322	0.532	0.978	0.883	0.476	0.635
O_3_	89.191	90.844	22.414	33.619	89.900	93.202	30.391	50.887
T_3_								
PM_2.5_	38.017	41.341	10.110	15.579	32.576	31.528	12.672	22.063
PM_10_	76.710	72.908	20.818	23.122	74.556	69.934	33.102	42.840
SO_2_	11.712	8.900	8.651	4.860	12.165	6.224	15.402	7.889
NO_2_	40.632	41.365	7.620	10.244	37.552	36.147	9.682	14.688
CO	1.094	1.132	0.417	0.599	0.964	0.842	0.506	0.707
O_3_	75.246	63.741	26.378	41.941	90.232	93.179	31.627	57.148
GB3095-2012 Air quality standards								
	Level I standard				Level II standard			
PM_2.5_	15				35			
PM_10_	40				70			
SO_2_	20				60			
NO_2_	40				40			
CO	4				4			
O_3_	160				200			

### Air pollutants and meteorological factors analysis for the Baota District

Based on Table 3 and table S3,We found that among the six pollutants, Only O_3_ is High positive correlation with temperature.The remaining five pollutants are negative correlation with meteorological factors, where SO_2_ and NO_2_ have no correlation with wind speed.SO_2_, CO and temperature are Moderate negative correlation, and PM_10_ is negligible correlation. PM_10_ and NO_2_ are Low negative correlation with relative humidity, and PM_2.5_, SO_2_, CO, O_3_ and relative humidity are negligible correlation. All air pollutants have a negligible correlation with wind speed.The mean value of temperature is 10.11 °C with a SD of 10.05 °C. The mean value of wind speed is 2.03 m/s with a SD of 0.81m/s. The mean value of relative humidity is 59.33% with a SD of 20.49%.

**Table 3:** Correlation analysis between atmospheric pollutants and meteorological factors

	PM_2.5_	PM_10_	SO_2_	NO_2_	CO	O_3_
Temperature	-0.385^**^	-0.158^**^	-0.525^**^	-0.402^**^	-0.564^**^	0.761^**^
Relative humidity	-0.109^**^	-0.405^**^	-0.294^**^	-0.408^**^	-0.124^**^	-0.078^**^
Wind speed	-0.070^**^	-0.025^**^	-0.012	-0.034	-0.019^**^	0.098^**^

^**^P < 0.01

### Logistic regression model and sensitivity analysis

Based on Table 4, before adjustment, all air pollutants except O_3_ were shown to be risk factors for preterm birth throughout pregnancy, early pregnancy and mid-pregnancy. After adjusting for maternal occupation, maternal parturition, last menstrual season, regularity of menstrual cycle, fetal birth weight, pregnancy complications, hypertension in pregnancy and meteorological factors, PM_2.5_ (OR: 1.098, 95% CI: 1.054–1.145), PM_10_ (OR: 1.031, 95% CI: 1.017–1.045), SO_2_ (OR: 1.107, 95% CI: 1.075–1.139), and NO_2_ (OR: 1.107, 95% CI: 1.060–1.156) exhibited risk factors for preterm birth throughout pregnancy. It means that for every 10 μg/m^3^ increase in pollutants, the risk of preterm birth increased by 9.8%, 3.1%, 10.7%, and 10.7%, respectively. In early pregnancy PM_2.5_ (OR: 1.049, 95% CI: 1.021–1.077), SO_2_ (OR: 1.053, 95% CI: 1.026–1.080), and NO_2_ (OR: 1.054, 95% CI: 1.015–1.094) behaved as a risk factor for preterm birth, and for every 10 μg/m^3^ increase in pollutants, the risk increased by 4.9%, 5.3% and 5.4%, respectively. In mid-pregnancy PM_2.5_ (OR: 1.045, 95% CI: 1.022–1.069), PM_10_ (OR: 1.018, 95% CI: 1.010–1.025), SO_2_ (OR: 1.088, 95% CI: 1.061–1.117) and NO_2_ (OR: 1.075, 95% CI: 1.039- 1.112) were risk factors for preterm birth, and the risk of preterm birth increased by 4.5%, 1.8%, 8.8% and 7.5%, respectively, when each 10 μg/m^3^ increase in pollutants was observed. In late pregnancy, PM_2.5_ (OR: 1.028, 95% CI: 1.005–1.052), PM_10_ (OR: 1.016, 95% CI: 1.009–1.023), SO_2_ (OR: 1.060, 95% CI: 1.040–1.080), NO_2_ (OR: 1.069, 95% CI: 1.037- 1.103) were all risk factors for preterm birth. When the pollutant mass concentration increased by every 10 μg/m^3^, the risk of preterm birth occurrence increased by 2.8%, 1.6%, 6%, and 6.9%. Exposure to CO was a risk factor for preterm birth throughout pregnancy, early, mid and late pregnancy (Table 4).

**Table 4:** Analysis of the correlation between air pollutants and preterm birth

	Unadjusted					Adjusted			
	Pollutants	P	OR	95%CI		P	OR	95%CI	
E	PM_2.5_	< 0.001	1.122	1.093	1.152	< 0.001	1.098	1.054	1.145
	PM_10_	< 0.001	1.050	1.040	1.060	< 0.001	1.031	1.017	1.045
	SO_2_	< 0.001	1.093	1.073	1.113	< 0.001	1.107	1.075	1.139
	NO_2_	< 0.001	1.057	1.027	1.088	< 0.001	1.107	1.060	1.156
	CO	< 0.001	3.987	2.571	6.184	< 0.001	7.645	3.705	15.773
	O_3_	< 0.001	0.971	0.960	0.982	< 0.001	0.941	0.921	0.962
T_1_	PM_2.5_	< 0.001	1.088	1.073	1.102	< 0.001	1.049	1.021	1.077
	PM_10_	< 0.001	1.015	1.01	1.020	0.565	1.002	0.994	1.011
	SO_2_	< 0.001	1.097	1.077	1.118	< 0.001	1.053	1.026	1.080
	NO_2_	< 0.001	1.124	1.101	1.147	0.006	1.054	1.015	1.094
	CO	< 0.001	10.340	7.159	14.934	< 0.001	5.171	2.776	9.633
	O_3_	< 0.001	0.968	0.964	0.973	0.001	0.981	0.971	0.992
T_2_	PM_2.5_	< 0.001	1.045	1.033	1.057	< 0.001	1.045	1.022	1.069
	PM_10_	< 0.001	1.027	1.022	1.032	< 0.001	1.018	1.010	1.025
	SO_2_	< 0.001	1.065	1.050	1.080	< 0.001	1.088	1.061	1.117
	NO_2_	< 0.001	1.031	1.014	1.049	< 0.001	1.075	1.039	1.112
	CO	< 0.001	2.380	1.748	3.241	< 0.001	5.453	2.936	10.128
	O_3_	0.578	1.001	0.997	1.006	< 0.001	0.970	0.959	0.981
T_3_	PM_2.5_	< 0.001	0.946	0.936	0.956	0.019	1.028	1.005	1.052
	PM_10_	0.06	0.995	0.991	1.000	< 0.001	1.016	1.009	1.023
	SO_2_	0.344	1.005	0.995	1.016	< 0.001	1.060	1.040	1.080
	NO_2_	< 0.001	0.944	0.93	0.957	< 0.001	1.069	1.037	1.103
	CO	< 0.001	0.402	0.304	0.532	< 0.001	4.663	2.616	8.312
	O_3_	< 0.001	1.023	1.019	1.027	0.285	0.995	0.985	1.004

Adjusted for Occupation, Number of births,Last menstrual season,Menstrual cycle,Fetal birth weight,Complications diseases,Hypertension in pregnancy,meteorological factors

### Sensitivity analysis

From Table 5,after adding each confounding factor one by one, the correlation between each pollutant and preterm birth did not change significantly, proving that the main model was stable (Table 5).

**Table 5:** Sensitivity analysis

	PM_2.5_	PM_10_	SO_2_	NO_2_	CO	O_3_
E						
Model1	1.101*	1.037*	1.098*	1.058*	3.321*	0.950*
Model2	1.096*	1.031*	1.116*	1.122*	8.664*	0.934*
Model3	1.092*	1.030*	1.112*	1.115*	7.849*	0.937*
Original model	1.098*	1.031*	1.107*	1.107*	7.645*	0.941*
T_1_						
Model1	1.059*	1.009*	1.053*	1.058*	4.624*	0.971*
Model2	1.037*	1.003	1.056*	1.051*	4.653*	0.979*
Model3	1.037*	1.003	1.054*	1.047*	4.389*	0.979*
Original model	1.049*	1.002	1.053*	1.054*	5.171*	0.981*
T_2_						
Model1	1.059*	1.022*	1.078*	1.032*	4.352*	0.983*
Model2	1.045*	1.017*	1.098*	1.069*	6.802*	0.973*
Model3	1.043*	1.017*	1.094*	1.064*	6.194*	0.974*
Original model	1.045*	1.018*	1.088*	1.075*	5.453*	0.970*
T_3_						
Model1	0.979*	1.009*	1.057*	0.995	1.816*	1.013*
Model2	1.022*	1.016*	1.067*	1.079*	4.883*	0.996
Model3	1.023*	1.016*	1.064*	1.077*	4.630*	0.994
Original model	1.028*	1.016*	1.060*	1.069*	4.663*	0.995

Original model adjusted for Occupation, Number of births,Last menstrual season,Menstrual cycle,Fetal birth weight,Complications diseases,Hypertension in pregnancy,meteorological factors *P < 0.05

### Analysis of the correlation between mixed pollutants and preterm birth

According to Table 6, thro0ughout pregnancy, mixing of PM_10_ with NO_2_ was associated with preterm birth (OR: 1.025, 95% CI: 1.009–1.040), and mixing of PM_10_ with O_3_ was also associated with the occurrence of preterm birth (OR: 1.027, 95% CI: 1.012–1.042).Mixing of SO_2_ with NO_2_, CO, and O_3_, respectively, was also correlated with preterm birth, with ORs and confidence intervals of (OR: 1.187, 95% CI: 1.125–1.252), (OR: 1.287, 95% CI: 1.172–1.413), and (OR: 1.098, 95% CI: 1.062–1.135), respectively. Mixing of NO_2_ with O_3_ was also correlated with preterm birth (OR: 1.061, 95% CI: 1.005–1.119) (Table 6).

**Table 6:** Analysis of the correlation between the two-pollutant model and preterm birth for E

E	P	OR	95%CI	
PM_2.5_ + SO_2_	0.283	0.973	0.926	1.023
PM_2.5_ + NO_2_	0.226	1.037	0.978	1.099
PM_2.5_ + CO	0.575	0.984	0.928	1.042
PM_2.5_ + O_3_	0.063	1.058	0.997	1.123
PM_10_ + SO_2_	0.444	1.006	0.990	1.023
PM_10_ + NO_2_	0.002	1.025	1.009	1.040
PM_10_ + CO	0.097	1.014	0.997	1.032
PM_10_ + O_3_	< 0.001	1.027	1.012	1.042
SO_2_ + NO_2_	< 0.001	1.187	1.125	1.252
SO_2_ + CO	< 0.001	1.287	1.172	1.413
SO_2_ + O_3_	< 0.001	1.098	1.062	1.135
NO_2_ + CO	0.069	0.922	0.844	1.006
NO_2_ + O_3_	0.031	1.061	1.005	1.119
O_3_ + CO	0.989	1.000	0.979	1.021

Adjusted confounders were maternal occupation, parity, season of last menstrual period, menstrual cycle, birth weight, pregnancy complications, gestational hypertension

From Table 7, in early pregnancy, mixing of PM_2.5_ with SO_2_ was associated with preterm birth (OR: 1.032, 95% CI: 1.005–1.060). PM_2.5_ was also associated with preterm birth by mixing with NO_2_ (OR: 1.040, 95% CI: 1.009–1.072). PM_2.5_ was also correlated with preterm birth by mixing with O_3_ (OR: 1.043, 95% CI: 1.017–1.070). Mixing of SO_2_ with NO_2_ was correlated with preterm birth (OR: 1.055, 95% CI: 1.019–1.093). Mixing of SO_2_ with O_3_ was also correlated with preterm birth (OR: 1.049, 95% CI: 1.023–1.075). Mixing of NO_2_ with O_3_ was correlated with preterm birth (OR: 1.044, 95% CI: 1.005–1.085) (Table 7).

**Table 7:** Analysis of the correlation between the two-pollutant model and preterm birth for T_1_

T_1_	P	OR	95%CI	
PM_2.5_ + SO_2_	0.019	1.032	1.005	1.060
PM_2.5_ + NO_2_	0.011	1.040	1.009	1.072
PM_2.5_ + CO	0.538	1.010	0.979	1.042
PM_2.5_ + O_3_	0.001	1.043	1.017	1.070
SO_2_ + NO_2_	0.003	1.055	1.019	1.093
SO_2_ + CO	0.790	1.005	0.969	1.043
SO_2_ + O_3_	< 0.001	1.049	1.023	1.075
NO_2_ + CO	0.085	0.953	0.902	1.007
NO_2_ + O_3_	0.028	1.044	1.005	1.085
O_3_ + CO	0.707	0.998	0.988	1.008

Adjusted confounders were maternal occupation, parity, season of last menstrual period, menstrual cycle, birth weight, pregnancy complications, gestational hypertension

We explain below for the Table 8,at mid-pregnancy, PM_10_ mixed with NO_2_, CO, and O_3_, respectively, was correlated with preterm birth with ORs and confidence intervals of (OR: 1.014, 95% CI: 1.006–1.022), (OR: 1.011, 95% CI: 1.003–1.020), and (OR: 1.015, 95% CI: 1.007–1.023).The mixing of SO_2_ with NO_2_ (OR: 1.096, 95% CI: 1.059–1.133), CO (OR: 1.123, 95% CI: 1.071–1.177), and O_3_ (OR: 1.074, 95% CI: 1.045–1.103) was associated with preterm birth (Table 8).

**Table 8:** Analysis of the correlation between the two-pollutant model and preterm birth for T_2_

T_2_	P	OR	95%CI	
PM_2.5_ + SO_2_	0.067	0.981	0.961	1.001
PM_2.5_ + NO_2_	0.850	0.997	0.971	1.025
PM_2.5_ + CO	0.188	0.982	0.957	1.009
PM_2.5_ + O_3_	0.434	1.012	0.983	1.041
PM_10_ + SO_2_	0.081	1.007	0.999	1.015
PM_10_ + NO_2_	0.001	1.014	1.006	1.022
PM_10_ + CO	0.005	1.011	1.003	1.020
PM_10_ + O_3_	< 0.001	1.015	1.007	1.023
SO_2_ + NO_2_	< 0.001	1.096	1.059	1.133
SO_2_ + CO	< 0.001	1.123	1.071	1.177
SO_2_ + O_3_	< 0.001	1.074	1.045	1.103
NO_2_ + CO	0.262	0.971	0.923	1.022
NO_2_ + O_3_	0.077	1.041	0.996	1.088
O_3_ + CO	0.070	1.011	0.999	1.022

Adjusted confounders were maternal occupation, parity, season of last menstrual period, menstrual cycle, birth weight, pregnancy complications, gestational hypertension

Based on Table 9, in late pregnancy, PM_10_ mixed with NO_2_ (OR: 1.009, 95% CI: 1.002–1.016) and with CO (OR: 1.009, 95% CI: 1.002–1.016) was associated with preterm birth. SO_2_ mixed with NO_2_ (OR: 1.044, 95% CI: 1.020–1.069) and with CO (OR: 1.053, 95% CI: 1.021–1.087) was associated with preterm birth (Table 9).

**Table 9:** Analysis of the correlation between the two-pollutant model and preterm birth for T_3_

T_3_	P	OR	95%CI	
PM_2.5_ + SO_2_	0.306	0.987	0.963	1.012
PM_2.5_ + NO_2_	0.502	0.991	0.966	1.017
PM_2.5_ + CO	0.246	0.984	0.958	1.011
PM_10_ + SO_2_	0.068	1.007	1.000	1.014
PM_10_ + NO_2_	0.017	1.009	1.002	1.016
PM_10_ + CO	0.011	1.009	1.002	1.016
SO_2_ + NO_2_	< 0.001	1.044	1.020	1.069
SO_2_ + CO	0.001	1.053	1.021	1.087
NO_2_ + CO	0.525	1.015	0.969	1.064

Adjusted confounders were maternal occupation, parity, season of last menstrual period, menstrual cycle, birth weight, pregnancy complications, gestational hypertension

### Lagging and cumulative effects of air pollutants

Across exposure windows, we observed a correlation between Lag1 and Lag2 in the lag window and Lag-2 in the cumulative window with preterm birth. In the lag window, for every 10 ug/m^3^ increase in PM_2.5_ mass concentration, the risk of preterm birth occurrence increased by 0.7% (95% CI: 1.001–1.014), 0.6% (1.001–1.012), respectively. In the Lag-2 cumulative window, the risk of preterm birth increased by 0.8% (1.001–1.015) for every 10 ug/m^3^ increase in PM_2.5_ mass concentration. No correlation between the other exposure windows and preterm birth was observed.

In the lag and cumulative study of PM_10_ and preterm birth, we found that Lag2, Lag5, Lag6, and Lag7 in the lag window were all correlated with preterm birth, and the magnitude of the change was not consistent across days. The strongest correlation was found on day 7 of the lag, with a 0.5% increase in the risk of preterm birth for every 10 ug/m^3^ increase in PM_10_ exposure (95% CI: 1.001–1.008). And the smallest correlation was found on day 2 of the lag, with a 0.2% increase in the risk of preterm birth for every 10 ug/m^3^ increase in PM_10_ exposure (95% CI: 1.001–1.004). The cumulative effect showed that the correlation between PM_10_ exposure mass concentration and preterm birth increased progressively with increasing number of days. The cumulative effect was strongest on cumulative day 7, with a 0.5% (95% CI: 1.002–1.008) increase in the risk of preterm birth occurring for each 10 ug/m^3^ increase in PM_10_ exposure.

In the lag and cumulative studies of SO_2_ and preterm birth, we found that both the lag and cumulative windows were correlated with the occurrence of preterm birth. The correlation increased gradually from day 0 to day 4 of the lag period. The strongest correlation was found on day 6, with a 2.2% (95% CI: 1.011–1.033) increase in the risk of preterm birth for every 10 ug/m^3^ increase in SO_2_ exposure. The lowest correlation was found on day 0 of the lag, with a 1.1% (95% CI: 1.003–1.019) increase in the risk of preterm birth for every 10 ug/m^3^ increase in SO_2_ exposure. In the cumulative effect, the correlation increased progressively as the cumulative number of days increased. The strongest correlation was found on cumulative day 7, with a 2.1% (95% CI: 1.010–1.033) increase in the risk of preterm birth for every 10 ug/m^3^ increase in SO_2_ exposure.

In the study of lag and cumulative effects of NO_2_ and preterm birth, we observed a correlation between lag day 1 to lag day 7 and an increased risk of preterm birth. The correlation gradually increased from lag day 1 to lag day 3 but weakened on lag day 4 and lag day 6. The strongest correlation was seen at lag day 5, with a 2.1% (95% CI: 1.009–1.032) increase in the risk of preterm birth for every 10 ug/m^3^ increase in NO_2_ exposure. The lowest correlation was seen at lag day 1, with a 1.2% (95% CI: 1.001–1.024) increase in the risk of preterm birth for every 10 ug/m^3^ increase in NO_2_ exposure. Cumulative effects showed that all windows were associated with preterm birth and increased with the number of cumulative days. The strongest effect was seen on cumulative day 7, with a 2.8% (95% CI: 1.012–1.043) increase in risk of preterm birth for every 10 ug/m^3^ increase in NO_2_ exposure.

In the study of lag and cumulative effects of CO and preterm birth, we observed that CO was correlated with preterm birth in both the lag window and the cumulative window. The risk of preterm birth was enhanced after maternal exposure to CO, but the correlation had different trends with lag days. The correlation was gradually increasing from day 0 to day 3 of the lag. The correlation was weakening from day 3 to day 6 of the lag. The strongest correlation was found on day 3, with a 59.2% (95% CI: 1.218–2.081) increase in the risk of preterm birth for every 10 mg/m^3^ increase in CO exposure. The weakest correlation was found on day 0 of the lag, with a 39.1% (95% CI: 1.070–1.809) increase in the risk of preterm birth for each 10 mg/m^3^ increase in CO exposure. The cumulative effect showed that the correlation was gradually increasing as the number of cumulative days increased. The strongest correlation was found on cumulative day 7, when the risk of preterm birth increased by 76.3% (95% CI: 1.288–2.413) for every 10 mg/m^3^ increase in CO exposure.

**Fig. 4:**
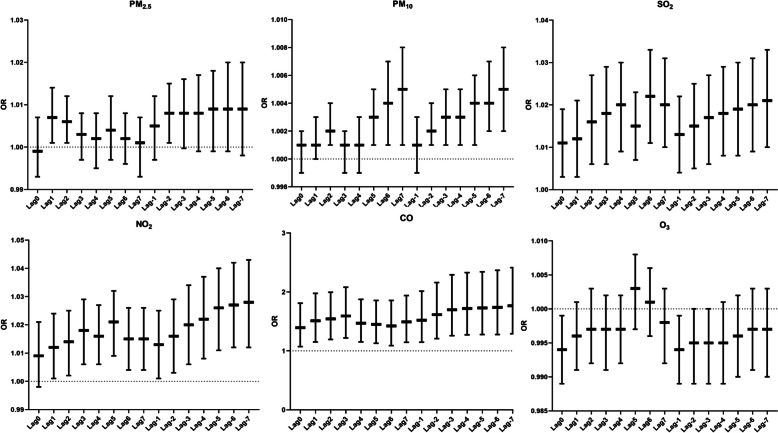
Lagged response and cumulative effects of air pollutants and preterm birth. Note: Adjusted confounders are maternal occupation, parity, season of last menstrual period, menstrual cycle, birth weight,pregnancy complications, pregnancy hypertension

In the study of lagged and cumulative effects of O_3_ and preterm birth, we did not find a correlation between pollutants and preterm birth in the lagged window. Pollutants were also not correlated with the occurrence of preterm birth in the cumulative window (Fig. 4).

## Relationship between contaminants and routine blood indicators

### Relationship between PM_2.5_ and human blood routine

**Fig. 5:**
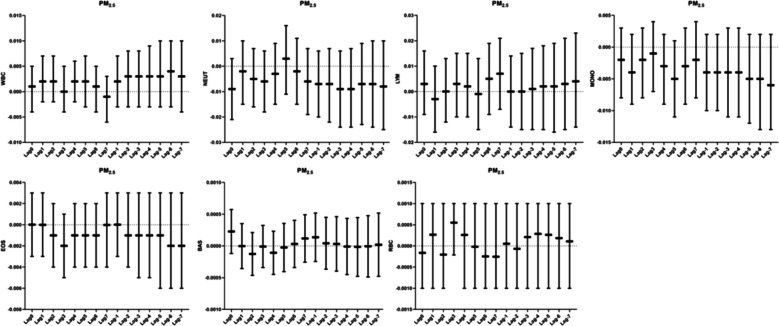
Correlation analysis between PM_2.5_ and blood routine

We did not observe any correlation of PM_2.5_ with leukocytes, neutrophils, lymphocytes, monocytes, eosinophils, basophils, and erythrocytes in the lag and accumulation windows (Fig. 5).

### Relationship between PM_10_ and human blood routine

**Fig. 6:**
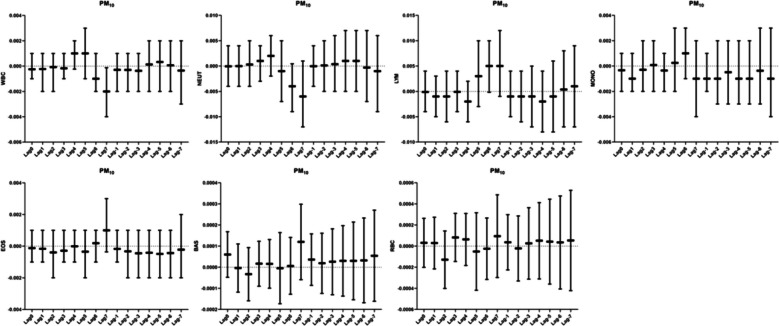
Analysis of the correlation between PM_10_ and blood routine

We found a correlation between pollutants and leukocytes on the day of Lag7 in our correlation analysis between PM_10_ and leukocytes. For every 10 ug/m^3^ increase in PM_10_ mass concentration, leukocytes decreased by 0.002 percentage points(95%CI:-0.004 ~ -0.0001). The rest of the window had no correlation with leukocytes. We also did not observe any correlation between PM_10_ and neutrophils, lymphocytes, monocytes, eosinophils, basophils, and erythrocytes in the lag and accumulation windows (Fig. 6).

### Relationship between SO_2_ and human blood routine counts

**Fig. 7:**
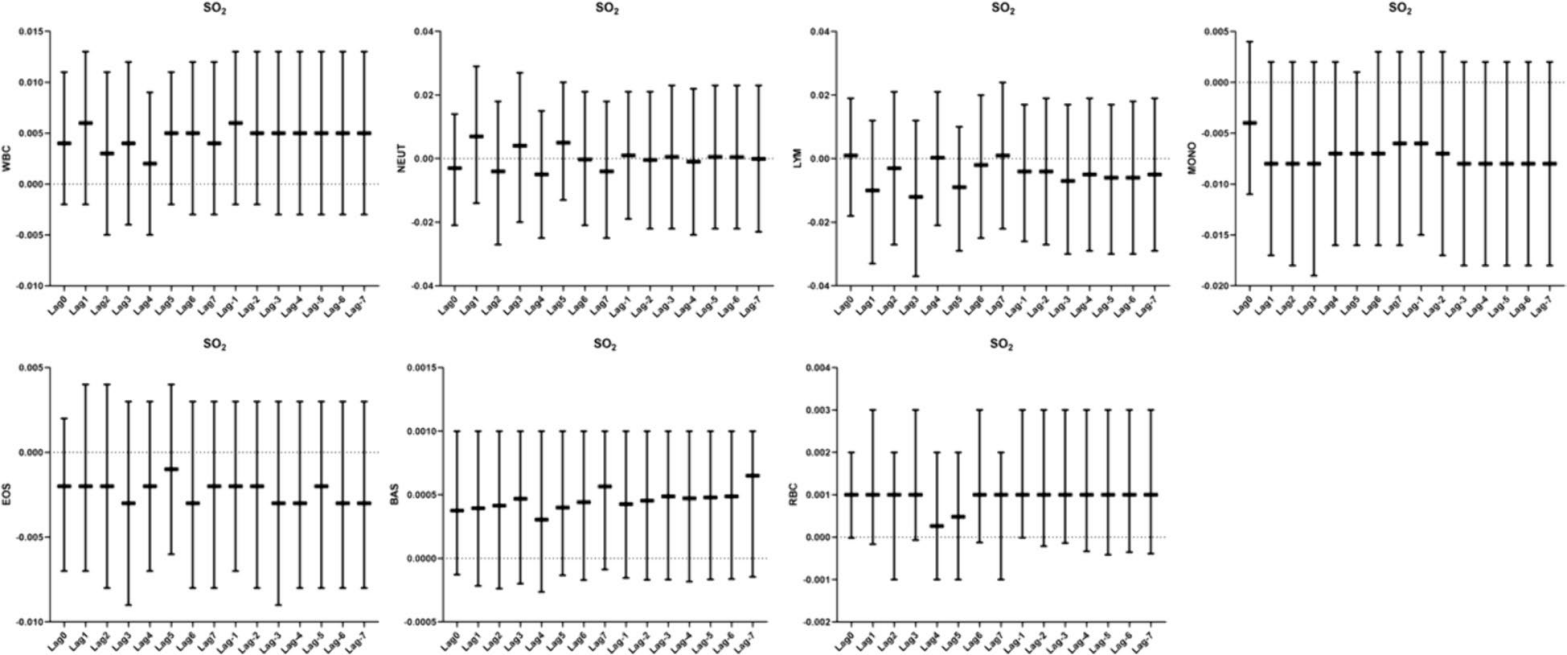
Analysis of the correlation between SO_2_ and blood routine

We did not observe a correlation between SO_2_ exposure and blood routine (Fig. 7).

### Relationship between NO_2_ and human blood routine

**Fig. 8:**
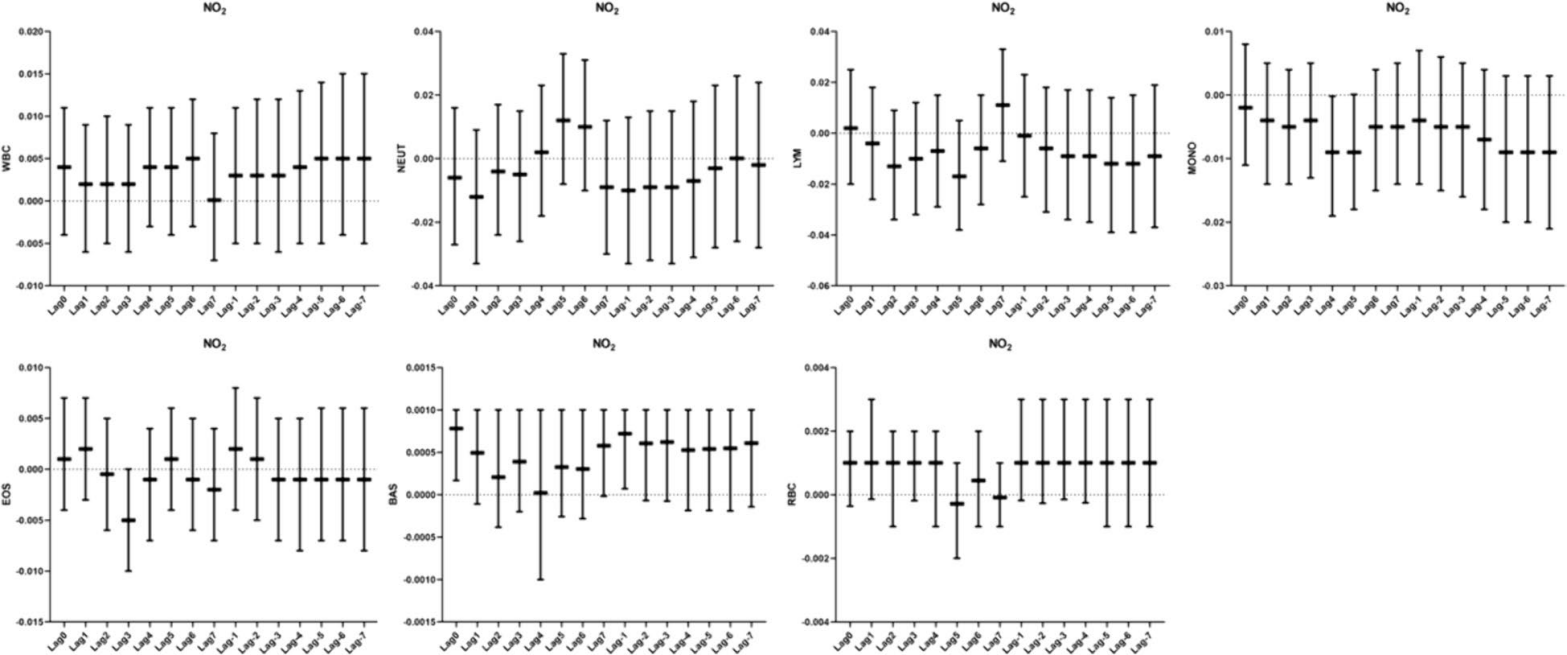
Analysis of the correlation between NO_2_ and blood routine

In the correlation analysis of NO_2_ with blood counts, we observed a correlation between NO_2_ and monocytes in the Lag4 exposure window, with monocytes decreasing by 0.009 percentage points (95% CI: -0.019 ~ -0.00018) for every 10 ug/m^3^ increase in NO_2_ mass concentration.NO_2_ correlates with basophils in Lag0 versus Lag-1. At both Lag0 and Lag-1, basophils were elevated by 0.001% for every 10 µg/m^3^ increase in NO_2_ mass concentration (Fig. 8).

### Relationship between CO and human blood routine

**Fig. 9:**
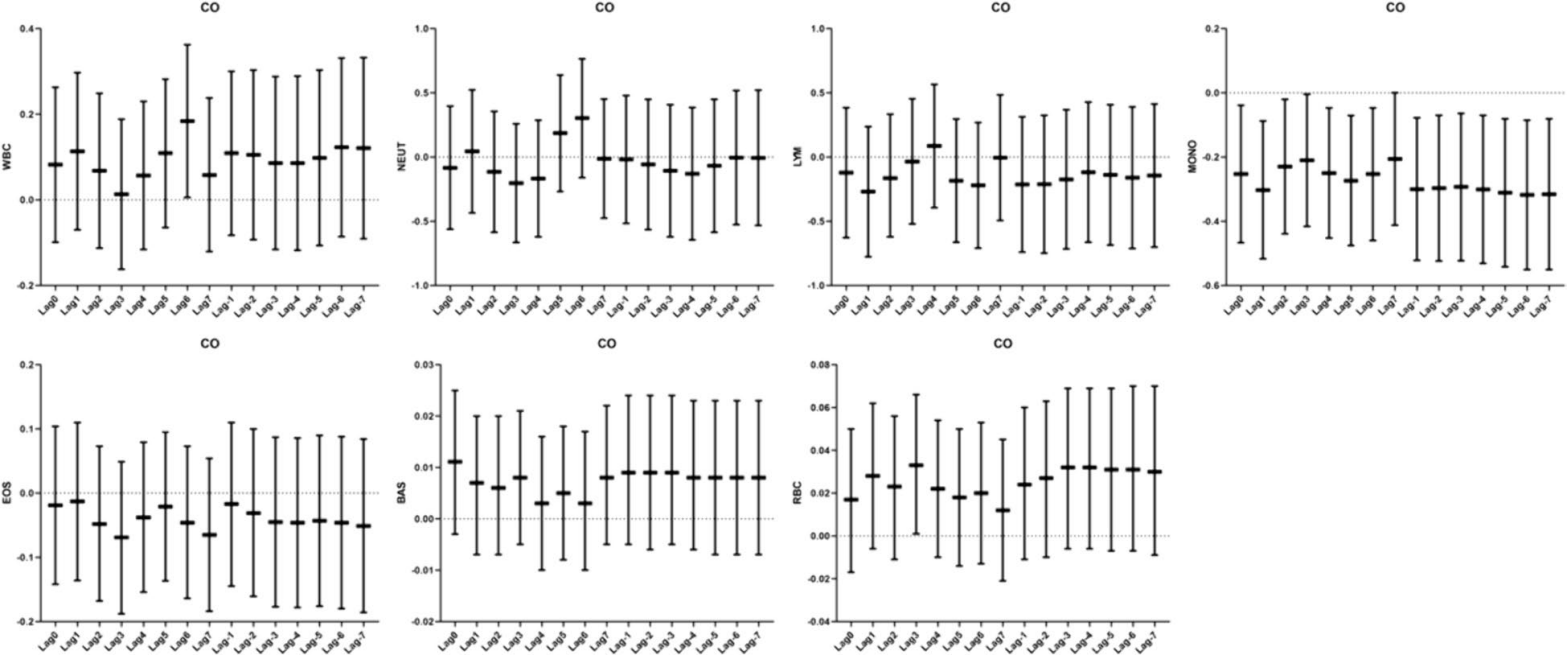
Analysis of the correlation between CO and blood routine

In the correlation analysis between CO and blood counts, we found that CO correlated with leukocytes in the Lag6 exposure window, with leukocytes rising by 0.184 × 10^9^/L (95%CI:0.006–0.362) for every 10 mg/m^3^ increase in CO mass concentration. Also,CO correlated with monocytes, with only Lag7 not observing a correlation throughout the exposure window. Monocytes decreased by 0.253%(95%CI:-0.467 ~ -0.039),0.303%(95%CI:-0.517 ~ -0.088), 0.23%(95%CI:-0.439 ~ -0.02),0.21%(95%CI:-0.416 ~ -0.004),0.25%(95%CI:-0.453 ~ -0.047),0.274%(95%CI:-0.476 ~ -0.071),and0.253%(95%CI:-0.460 ~ -0.047) for each 10 mg/m^3^ increase in CO mass concentration, respectively. The cumulative effect showed that monocytes decreased by 0.3%(95%CI:-0.522 ~ -0.078), 0.297%(95%CI:-0.524 ~ -0.07),0.293%(95%CI:-0.523 ~ -0.064), 0.301%(95%CI:-0.531 ~ -0.07),0.311%(95%CI:-0.542 ~ -0.081),0.318%(95%CI:-0.551 ~ -0.085),and0.316%(95%CI:-0.551 ~ -0.081), respectively, for every 10 mg/m^3^ increase in CO mass concentration.CO also correlated with erythrocytes. In the Lag-3 window, for every 10 mg/m^3^ increase in CO mass concentration, the erythrocytes increased by 0.033 × 10^12^/L(95%CI:0.001–0.066) (Fig. 9).

### Relationship between O_3_ and human blood routine

**Fig. 10:**
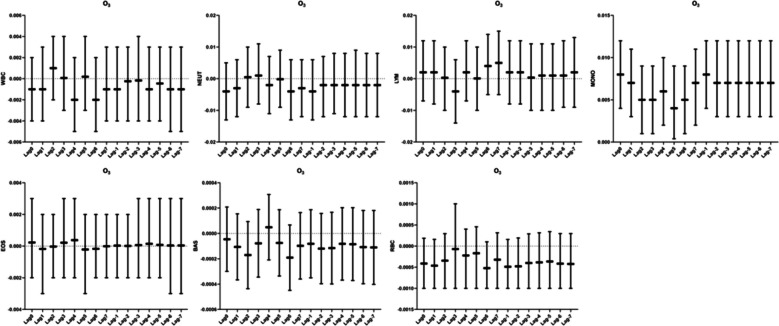
Analysis of the correlation between O_3_ and blood routine

In the O_3_ with blood correlation analysis, we only observed a correlation between exposure to O_3_ and monocytes. Every 10 mg/m^3^ increase in O_3_ mass concentration, monocytes increased by 0.008%(95%CI:0.004–0.012), 0.007%(95%CI:0.003–0.011), 0.005%(95%CI:0.001–0.009), 0.005%(95%CI:0.001–0.009), 0.006%(95%CI:0.002–0.01), 0.004%(95%CI:0.0004–0.009),0.005%(95%CI:0.001–0.009),0.007%(95%CI:0.002–0.011), respectively, and the cumulative effect indicated that for every 10 mg/m^3^ increase in O_3_ mass concentration, monocytes increased by 0.008% (95% CI: 0.004–0.012) at Lag-1 and by 0.007% (95% CI: 0.003–0.012) in each of the remaining cumulative windows (Fig. 10).

### C-reactive protein and ultrasensitive C-reactive protein and air pollutants

**Fig. 11:**
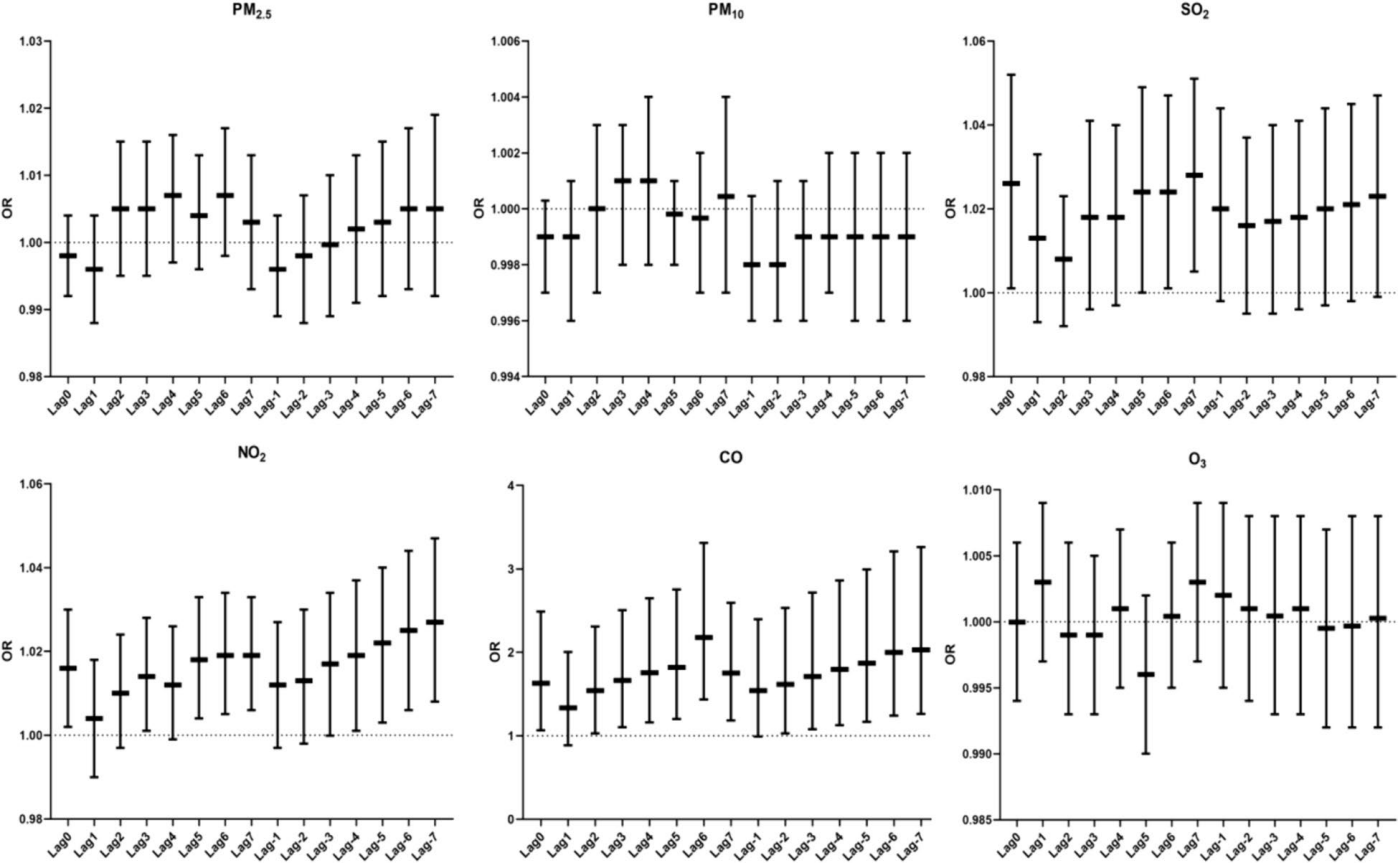
Correlation analysis between air pollutants and hs-CRP

In the study of the correlation between air pollutants and hs-CRP, we found that SO_2_, NO_2_, and CO were correlated with hs-CRP in certain windows (Fig. 10). SO_2_ was correlated with hs-CRP in the lag windows Lag0, Lag6, and Lag7, and the strongest lag window was the 7th day of lag (OR: 1.028, 95% CI: 1.005–1.051). NO_2_ correlated with hs-CRP in the lag window Lag0, Lag3, Lag5, Lag6, Lag7, with the strongest lag window being lag day 7 (OR: 1.019, 95% CI: 1.006–1.033). NO_2_ also correlated with hs-CRP in the cumulative window Lag-4, Lag-5, Lag-6, Lag-7, and the strongest correlation window was on cumulative day 7 (OR: 1.027, 95% CI: 1.008–1.047).CO correlated with hs-CRP in most of the exposure windows of lag and cumulative, and the correlation was increasing with the increase in the number of lag days in the lag window. The strongest correlation was reached at lag day 6 (OR: 2.181, 95% CI: 1.436–3.311). In the cumulative window, the correlation was gradually increasing as the cumulative days increased. The strongest correlation was reached on cumulative day 7 (OR: 2.028, 95% CI: 1.262–3.260) (Fig. 11).

**Fig. 12:**
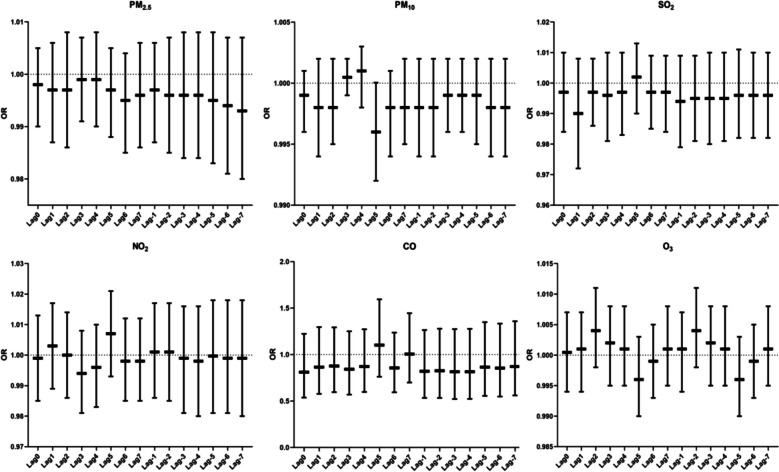
Correlation analysis between air pollutants and CRP

We did not observe a correlation between air pollutants and CRP across exposure windows (Fig. 12).

## Discussion

In this study, the data of pregnant women with preterm birth in two tertiary hospitals in Baota District, Yan'an City from January 2018 to December 2020 were collected. Conditional logistic regression model was used to investigate the relationship between air pollutants and preterm birth and it was found that PM_2.5_ was associated with preterm birth during the whole pregnancy period, early pregnancy period, mid-pregnancy period and late pregnancy period and PM_10_ was associated with preterm birth during the whole pregnancy period, mid-pregnancy period and late pregnancy period and SO_2_ was associated with the increased risk of preterm birth during the four windows. NO_2_ was associated with preterm birth throughout pregnancy. Exposure to CO during pregnancy was associated with preterm birth. O_3_ was not observed to be associated with preterm birth in this study. In short-term lagged and cumulative effects, PM_2.5_, PM_10_, SO_2_, NO_2_ and CO were correlated with increased risk of preterm birth in lagged effects; in cumulative effects, PM_2.5_, PM_10_, SO_2_, NO_2_ and CO were also correlated with increased risk of preterm birth. We also observed that SO_2_, NO_2_ and CO were correlated with hs-CRP in some windows, and no correlation between air pollutants and CRP was observed.

Xiaotong et al. study on PM_2.5_ and preterm birth in Wuhan found that exposure to PM_2.5_ during pregnancy was associated with the occurrence of preterm birth in all four windows as well [33], Which is consistent with our findings. Ju et al. found a correlation between PM_10_ and preterm birth in late pregnancy and throughout pregnancy, which is similar to our findings [34]. He et al. showed a correlation between SO_2_ and preterm birth in mid- and late-pregnancy, which is similar to our results [35]. A study of short-term exposure to air pollutants in preterm birth in Xi'an, China, demonstrated a correlation between SO_2_ and preterm birth [36]. Some studies have found that exposure to high levels of CO in early pregnancy and throughout pregnancy leads to an increased risk of very preterm birth [37], this is similar to our findings. Su et al. showed that PM_10_ in the first 3 months of pregnancy was associated with preterm birth, and the study also demonstrated a lag between PM_2.5_ and PM_10_ on preterm birth [38].

There are also some studies that differ from our results, Sheridan et al. noted that PM_2.5_ was associated with preterm birth throughout pregnancy, weeks 17–24 of gestation, and week 36 of gestation PM_2.5_, which is inconsistent with our results [39]. Chen et al. showed a correlation between SO_2_ in late pregnancy and preterm birth [40], no association was found between SO_2_ and preterm birth in early and mid-pregnancy. Zhou et al. showed that SO_2_ and NO_2_ was not associated with preterm birth [41], which is inconsistent with our findings.A study of air pollutants and preterm birth in California showed that exposure to O_3_ was associated with an increased incidence of preterm birth, and also found that the hysteresis effect of O_3_ could also increase the incidence of preterm birth [42], which is inconsistent with our results.

Regarding the study of air pollutants and blood routines, Pilz et al. showed similar results to ours in that they found that PM_10_, PM_2.5_, NO_2_, and NO_X_ one-pollutant models showed a positive, but not significant, correlation with hs-CRP [43]. Kim et al. showed that hs-CRP was correlated with PM_2.5_, PM_10_, SO_2_, and NO_2_, which is similar to our findings, but the exposure windows in that study were all long-term exposures [44]. Tang et al. showed a correlation between exposure to air pollutants and increased CRP levels in COPD patients the day before hospitalization, which is inconsistent with our results [45]. Liu et al. similarly showed that exposure to air pollutants was associated with increased circulating CRP levels, which is inconsistent with our results [46]. Gogna et al. showed that exposure to air pollution was associated with abnormal CRP levels, which is different from our results [47].

Regarding these inconsistent results, we found that the possible reasons are as follows. Firstly, the variability of the study areas: different areas have their own unique geomorphology and pollutant sources. Secondly, different experimental designs: different exposure windows and different exposure assessment methods may cause variations in the results. Finally, there are differences in climatic characteristics: climate is the main factor affecting air pollutants, and each study area has its own unique climatic characteristics, which leads to differences in the distribution of air pollutants.

While the data from the four pollutant monitoring stations used in this study allow for a relatively accurate assessment of exposure to individual pollutants. However, the pollutant exposure levels for each window period in the study were based on the residential address during pregnancy, a method that only considers outdoor exposure and ignores the daily mobility of pregnant women, which may introduce bias and measurement error into the study. In addition, although the study populations we chose were all located in the Baota District, there will still be some study populations living farther away from the monitoring stations, and therefore the assessment of pollutant mass concentrations in these populations may not be accurate. Finally, some other unknown confounders may not have been collected in this study, so the results may be biased to some extent.

## Conclusion

In this study, we investigated the correlation between air pollutants and the occurrence of preterm birth in pregnant women in Baota District and the relationship between air pollutants and blood cell counts of pregnant women. The results of the study showed that in the long-term effect of air pollutants, pollutants were associated with preterm birth in different exposure windows. In lagged effects, PM_2.5_, PM_10_, SO_2_, NO_2_, and CO were associated with an increased risk of preterm birth; in cumulative effects, PM_2.5_, PM_10_, SO_2_, NO_2_, and CO were also associated with an increased risk of preterm birth. Correlation analysis between air pollutants and blood cell counts showed that exposure to PM_10_ was associated with changes in leukocyte counts, exposure to NO_2_ was associated with changes in monocyte and basophil counts, respectively, and exposure to CO was associated with changes in leukocyte, monocyte, and erythrocyte counts, respectively. Air pollutants were associated with hs-CRP in the lag and cumulative windows.

### Supplementary Information

Supplementary Material 1.

## Data Availability

The datasets used and/or analysed during the current study are available from the corresponding author on reasonable request.
