# Supplementary material for: The relationship between air pollutants and preterm birth and blood routine changes in typical river valley city
Source: BMC Public Health. 2024 Jun 24;24:1677. doi: 10.1186/s12889-024-19140-2 (PMC11197378; doi:10.1186/s12889-024-19140-2)
Supplement: Supplementary file 1 — Supplementary Material 1. [file 12889_2024_19140_MOESM1_ESM.docx]

| Table S1. Descriptive Index of Meteorological Factors in Baota District | | | | | |
| --- | --- | --- | --- | --- | --- |
|  | Mean | Median | Minimum | Maximum | SD |
| temperature(℃) | 10.11 | 10.95 | -15.10 | 28.30 | 10.05 |
| wind speed(m/s) | 2.03 | 1.90 | 0.30 | 5.90 | 0.81 |
| relative humidity(%) | 59.33 | 59.00 | 15.00 | 99.00 | 20.49 |
